# Turkish version of a short nutrition literacy scale for young adults: cultural adaptation and validation

**DOI:** 10.3389/fnut.2024.1422738

**Published:** 2024-07-22

**Authors:** Mustafa Can Koc, Elif Yildirim, Ozkan Isik, Pelin Aksen, Laurentiu-Gabriel Talaghir, Ciprian Zanfir

**Affiliations:** ^1^School of Physical Education and Sports, Istanbul Gelisim University, Istanbul, Türkiye; ^2^Directorate of Sports Sciences Application and Research Center, Istanbul Gelisim University, Istanbul, Türkiye; ^3^Department of Statistics and Quality Coordinator, Konya Technical University, Konya, Türkiye; ^4^Faculty of Sports Sciences, Balıkesir University, Balikesir, Türkiye; ^5^Directorate of Sports Sciences Application and Research Center, Balikesir University, Balikesir, Türkiye; ^6^Faculty of Sports Sciences, Kırıkkale University, Kırıkkale, Türkiye; ^7^Faculty of Physical Education and Sport, Dunarea de Jos University of Galati, Galati, Romania

**Keywords:** cultural adaptation, food consumption, nutritional behavior, nutrition literacy, reliability

## Abstract

**Background:**

Recent changes in nutrition trends may lead to health issues. In particular, the unhealthy eating habits of young adults suggest that future generations may be at risk. Therefore, the importance and necessity of nutrition literacy is becoming increasingly apparent.

**Purpose:**

The purpose of this study was to create a Turkish version of a short nutrition literacy (S-NutLit-Tr) scale for young adults developed by Vrinten et al (2023).

**Methods:**

This research involved 115 young adults from Istanbul Gelişim University, who were selected through convenience sampling, a non-probability sampling method. After the original S-NutLit scale was translated into Turkish, the validity and reliability of the determined factor structure were tested on 115 young adults. Internal consistency was examined with the Cronbach Alpha coefficient. Confirmatory factor analysis was employed to validate the two sub-scale structures. Finally, measurement invariance was tested concerning participants’ gender, aiming to ascertain whether the scale captured equivalent characteristics across different groups.

**Results:**

As a result of the reliability analysis conducted with the scale of S-NutLit-Tr, the Cronbach Alpha coefficient was obtained as 0.86 for the scale of S-NutLit-Tr. Additionally, it was found to be 0.84 and 0.77 for the “information skills” and “expert skills” sub-scales, respectively. Accordingly, the scale of S-NutLit-Tr was found to be reliable. To examine the two sub-scale factor structures of the S-NutLit-Tr scale, fit indices were examined: χ^2^/df (1.246), GFI (0.923), IFI (0.975), TLI (0.967), CFI (0.974), RMSEA (0.046), and SRMR (0.055) and it was observed that the indices were within acceptable limits. In the analysis results obtained through the multi-group confirmatory factor analysis for measurement invariance, it was observed that the ∆CFI and ∆TLI values across all indices were less than or equal to 0.01. Consequently, it was observed that the item-factor structure, factor loadings, variances, covariances, and error variances of the scale were equivalent for both male and female young adults.

**Conclusion:**

The study found that the scale of S-NutLit-Tr for young adults was a valid and reliable measurement tool in Turkish culture.

## 1 Introduction

One of the most important elements of a healthy life is nutrition (1, 2). Nutrition refers to the deliberate actions taken by individuals to consume the necessary nutrients in appropriate quantities and at the right time to enhance health, maintain current health status, and improve quality of life (3).

In order to make the most appropriate decisions in these actions and to develop healthy eating behaviors accordingly, individuals should improve their knowledge of the scientific evidence base regarding nutrition (4). Nutrition information is defined as the knowledge of all concepts and processes related to nutrition and health. This includes an understanding of the effect of diet on health, the relationship of diet with diseases, the identification of foods that represent basic nutrients, the formulation of guidelines on nutrition, and the formulation of nutrition recommendations (5).

Research has shown that most people in Turkey possess basic nutrition knowledge. However, their understanding and attitudes toward selecting healthy foods, determining appropriate portion sizes, reading food labels, and being aware of nutrition values are lacking (6, 7). Studies have shown that individuals with sufficient nutrition knowledge often struggle to apply this knowledge in their daily lives (8, 9). For this reason, it is of great importance to introduce the measurement tool for nutrition literacy into Turkish culture. To promote balanced and adequate nutrition habits, prevent nutrition-related problems that reduce quality of life, and avoid nutrition-related chronic diseases, it is essential to assess the nutrition literacy of individuals in society. Depending on the results obtained through the measurement tool created, perhaps national projects, programs, and training on nutrition should be planned to increase nutrition literacy (10, 11).

Nutrition literacy is the capacity to access essential nutrition information and services required to improve or maintain an individual’s health status, comprehend, and interpret the information, and apply it in practice. This concept is examined in three sub-scales: functional, interactive, and critical nutrition literacy (12). Nutrition literacy encompasses the abilities required for individuals to correctly prepare and cook foods, demonstrate a healthy food selection attitude, and understand the effects of economic and environmental factors on their food choices (13). Short Nutrition Literacy (S-NutLit) focuses on young adults as a measurement tool. Emerging adulthood is the period between the ages of 18 and 25 when individuals have not yet fully transitioned into adulthood (14). This period is characterized by the maturation of the prefrontal cortex and the development of reasoning and self-regulation functions (15).

The prefrontal cortex in development has less capacity than the mature prefrontal cortex to control reward-oriented behaviors, such as consuming calorie-dense and highly palatable foods. Excessive consumption of calorie-dense foods may lead to permanent cognitive and behavioral changes. The physical harm caused by overconsumption of these foods may be concealed by the rapid growth spurt during adolescence. Adolescent neurodevelopment relies on good nutrition for optimal brain health (16). This information demonstrates the significance of researching the nutrition literacy of young adults. This research will reveal a measurement tool to determine the nutrition literacy required by young adults throughout their lives. This measurement tool will serve as a guide for orientation and training.

## 2 Methods

### 2.1 Adaptation to Turkish of the items

For this study, two bilingual (Turkish and English) researchers independently translated the original S-NutLit scale from English to Turkish. The scale’s language and content validity were assessed by five different experts. Each item was scored on a scale of 1–4, with 4 indicating appropriateness, 3 indicating appropriateness with minor suggested changes, 2 indicating somewhat appropriateness with major requested changes, and 1 indicating inappropriateness. The Content Validity Index (CVI) values for the scale were calculated using the Davis method. The CVI values are calculated by determining the proportion of experts who gave 4 and 3 points for the items on the scale, divided by the total number of experts. A criterion of 0.80 is accepted for the values (17). The final version of the scale was created by making necessary corrections based on expert opinions (see Appendix 1). The CVI values for language and content validity were both above 0.80. During the second stage, the reliability of the scale, which consisted of two factors and 11 items, was analyzed using Exploratory Factor Analysis (EFA) to determine the factor structures. Confirmatory Factor Analysis (CFA) was conducted in the third stage to examine the validity of the Turkish version of the scale, using the model obtained by EFA.

### 2.2 Participants

The study involved 115 young adults aged 18–25 who were studying at Istanbul Gelisim University. In the literature, there were various studies on determining the optimal sample size for factor analysis. The recommended method in these studies is to have at least 10 participants per item (18–20). The validity and reliability of the S-NutLit scale, which consists of 11 items, were tested on 115 young adults using this approach. The study’s participants were young adults selected through convenience sampling, a non-probabilistic sampling method, in line with the research topic.

### 2.3 Data collection tools

This study adapted the S-NutLit-Tr scale to determine the nutrition literacy of young adults aged 18–25 years. Data were collected using the S-NutLit scale. The scale comprises 11 items, each rated on a five-point Likert scale ranging from “completely agree (5)” to “completely disagree (1).” The factor analysis revealed two sub-dimensions: Information Skills (IS) (items 1–8) and Expert Skills (ES) (items 9–11). The original S-NutLit scale was validated and tested for reliability on young adults. The Cronbach’s Alpha value for the scale was 0.80, while the IS sub-scale had a value of 0.83 and the ES sub-scale had a value of 0.79.

### 2.4 Statistical analysis

The statistical analyses were conducted using SPSS 25.0 and R package version 4.0.2. To assess the language and content validity of the Turkish adapted scale, expert opinions were obtained and CVI values were analyzed. The Cronbach Alpha coefficient was used to calculate the internal consistency level of the scale. To ensure high reliability, the Cronbach Alpha value should be 0.70 or higher (17). The scale’s construct validity was tested using CFA, and the following indices were evaluated to assess how well the data fit the model: the ratio of chi-square to degrees of freedom (χ^2^/df), root mean square error of approximation (RMSEA), comparative fit index (CFI), standardized root mean square residual (SRMR), Tucker Lewis Index (TLI), Goodness of Fit Index (GFI), and Incremental Fit Index (IFI) (21–23). Furthermore, the study analyzed the scale’s measurement invariance regarding gender to determine if the scale measured the same characteristics across different groups.

### 2.5 Legal permission and ethical approval

Before conducting the Turkish adaptation study of the S-NutLit scale for young adults developed by Vrinten (1), we obtained the required legal permissions. In addition, we obtained the necessary ethical permissions from the Istanbul Gelisim University ethics committee (Meeting no: 2024-03, Meeting date: 29.02.2024).

## 3 Results

To test the measurement invariance of the S-NutLit-Tr scale, we collected gender demographic data from the young adults participating in the study. The results showed that 44.3% of the 115 participants were female and 55.7% were male.

### 3.1 Exploratory factor analysis

An EFA was carried out to assess the appropriateness of the factor structures in the original scale for Turkish culture. The suitability of the data for factor analysis was assessed by examining the overall Kaiser-Meyer-Olkin (KMO) value of the scale and Bartlett’s test of sphericity. The scale’s KMO measure was 0.87, and Bartlett’s test of sphericity was statistically significant [χ^2^(55) = 442.582, *p* < 0.0001]. The data were deemed appropriate for principal component analysis (PCA). The results showed that the original structure, consisting of two factors, remained unchanged in Turkish culture. The items were divided into two sub-scales: IS (items 1–8) and ES (items 9–11). Table 1 presents the component loadings of the solutions obtained using Varimax rotation.

**Table 1:** Factor loadings obtained by the Varimax rotation method.

Items	Factor 1 (IS)	Factor 2 (ES)
If I have questions about healthy nutrition, I know where I can find information about it.	0.611	
If I have questions about sustainable nutrition, I know where I can find information about it. Examples of sustainable nutrition are: organic vegetables, free-range eggs, fair trade coffee, etc.	0.700	
I am familiar with the basic rules of the Food Triangle.	0.565	
I can assess whether information about nutrition is written with the intention of making money, for example by people who want to sell a product.	0.648	
When I search for information about nutrition on the internet, I can distinguish between reliable and less reliable websites.	0.736	
Advertisements often link nutrition and health. I find it easy to judge whether these links are correct or not.	0.797	
I have the necessary skills to apply information about nutrition when cooking.	0.650	
I can assess whether information about nutrition in the media is reliable.	0.573	
I discuss information about nutrition with experts.		0.802
I follow nutritional advice from experts.		0.774
I base my diet on the latest scientific knowledge.		0.828

IS, Information skills; ES, Expert skills.

### 3.2 Confirmatory factor analysis

**Figure 1:**
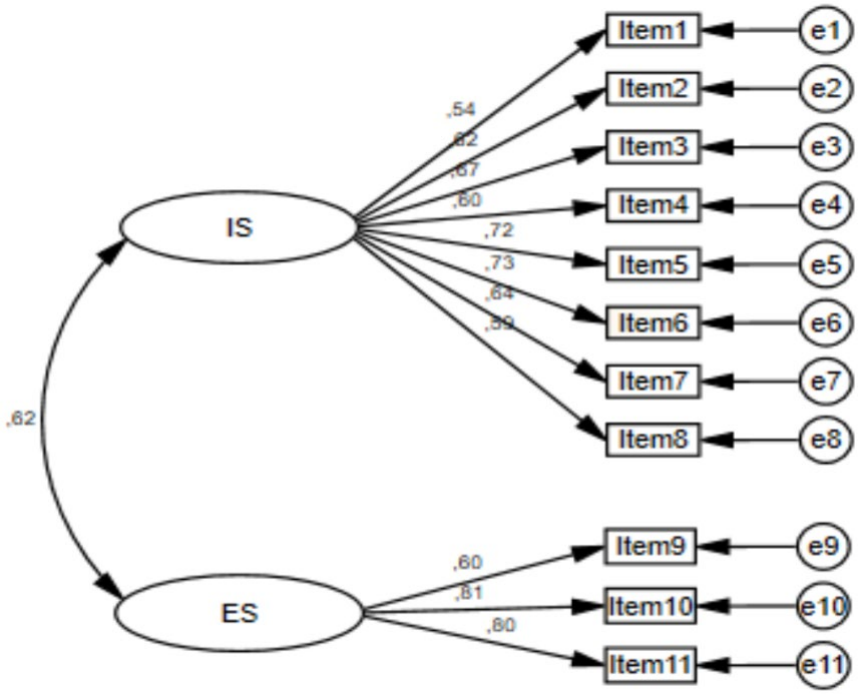
The CFA loading of S-NutLit-Tr. e1, e2, …, e11 show measurement errors.

Confirming the two-factor structure revealed by EFA, CFA analysis was conducted. The scale’s Turkish version, named S-NutLit-Tr, consists of 11 items. The Cronbach’s Alpha value for the entire scale was 0.86, while the IS subscale and ES subscale had Cronbach’s Alpha values of 0.84 and 0.77, respectively. Fit indices were calculated, including χ^2^/df (1.246), CFI (0.974), TLI (0.967), RMSEA (0.046), and SRMR (0.055). The study results confirmed the acceptability and applicability of the S-NutLit-Tr scale, as shown in Table 2 and Figure 1. The study tested the measurement invariance of the S-NutLit-Tr scale based on gender. The analysis followed the hierarchy of structural, metric, scale, and strict invariance. The study examined whether the measurement invariance between the two hierarchical levels was maintained by considering the ∆CFI and ∆TLI values. A difference of 0.01 or less in both ∆CFI and ∆TLI between the hierarchical rankings indicates that measurement invariance has been achieved at the relevant stage (24).

**Table 2:** Fit indices of the models for the Turkish form of the S-NutLit-Tr in CFA.

	S-NutLit-Tr	Acceptable fit	Perfect fit
CMIN/df	1.246	<5	<3
GFI	0.923	>0.90	>0.95
IFI	0.975	>0.90	>0.95
TLI	0.967	>0.90	>0.95
CFI	0.974	>0.90	>0.95
RMSEA	0.046	<0.08	<0.05
SRMR	0.055	<0.10	<0.05

CMIN/df, Minimum discrepancy of confirmatory factor analysis/Degrees of freedom; GFI, Goodness of fit index; IFI, Incremental fit index; TLI, Tucker-Lewis index; CFI, Comparative fit index; RMSEA, Root mean square error of approximation; SRMR, Standardized root mean squared residual.

Upon analysis of Table 3, it can be interpreted that all invariances are provided since the structural, metric, scale, and strict invariance findings according to gender have good fit values in both groups (RMSEA < 0.08, SRMR < 0.08, TLI, CFI > 0.90). It was observed that the differences in ∆CFI and ∆TLI values between metrics were less than or equal to 0.01. Full measurement invariance was achieved when analyzing the goodness-of-fit statistics using the multi-group CFA method based on gender. The item-factor structure, factor loadings, variances, covariances, and error variances of the S-NutLit-Tr scale are equivalent for both male and female young adults.

**Table 3:** Fit statistics for measurement invariance according to gender.

	χ^2^	df	χ^2^/df	CFI	TLI	RMSEA	SRMR	∆CFI	∆TLI
Configural	129.209	86	1.502	0.904	0.878	0.093	0.077	-	-
Metric	139.475	95	1.468	0.902	0.886	0.090	0.097	−0.003	0.004
Scalar	145.382	104	1.398	0.908	0.903	0.083	0.099	0.006	0.002
Strict	157.662	115	1.371	0.906	0.910	0.080	0.100	−0.002	0.007

χ^2^, Chi-square; df, Degrees of freedom; CFI, Comparative fit index; TLI, Tucker-Lewis index; RMSEA, Root mean square error of approximation; SRMR, Standardized root mean squared residual.

## 4 Discussion and conclusion

This research examined the validity and reliability of the “Short Nutrition Literacy Scale for Young Adults” developed by Vrinten et al. (1) to introduce the scale into Turkish literature. The scale was first translated into Turkish and its language and content validity were tested. The final version of the scale was formed with suggestions from experts. The construct validity was tested using EFA, which revealed that the items in the Turkish adaptation of the scale, originally consisting of two factors, were grouped into two factors. The factors were labeled as “IS” and “ES,” based on the content of the original scale and its suitability to the Turkish social structure. To evaluate the scale’s reliability, we calculated the Cronbach Alpha value, which was statistically significant. The two-factor structure was tested using CFA, and the fit index coefficients indicate that the scale fits well.

A multiple-group CFA analysis was conducted to test the measurement invariance of the scale among young adult males and females. The analyses revealed that young adults’ nutrition literacy demonstrated structural, metric, scale, and strict measurement invariance by gender. Accordingly, the means, variances, covariances, and error variances of the model were found to be equivalent for both female and male groups. The scores obtained from the nutrition literacy model can be compared between young adult male and female groups.

The study found that the S-NutLit-Tr scale was a valid and reliable tool for measuring the nutrition literacy of young Turkish adults. The scale consisted of a total of 11 items and two sub-dimensions as IS (items 1–8) and ES (items 9–11) and it was rated on a five-point Likert-type scale. The minimum and maximum scores to be obtained from the S-NutLit-Tr scale were between 11 and 55 points. High scores obtained from the S-NutLit-Tr scale indicate high nutrition literacy.

When previous studies on nutrition literacy were examined, Diamond (10) developed a 28-item initial measurement tool and found the Cronbach’s Alpha coefficient for this measurement tool to be 0.84 for adults. Subsequent studies continued to test the nutrition literacy scale developed by Diamond (10) in different populations and languages. For example, Patel et al. (25) reported that the nutrition literacy scale developed for adults was usable for Afro-American geriatric (>65 years old) patients. When adaption studies to different languages were examined, in the adaptation of the nutrition literacy scale to the Spanish language, Coffman and La-Rocque (26) found the Kuder–Richardson coefficient of reliability, which is a variant of the Cronbach’s coefficient alpha designed for scales with binary items, to be 0.95 and reported strong reliability for 134 Latino-American. Michou et al. (27) adapted the nutrition literacy scale to the Greek language in 50 adult individuals and reported that it would be a useful tool in assessing nutrition literacy. Sharifnia et al. (28) found the Cronbach’s Alpha coefficient for the Persian version of the nutrition literacy scale to be 0.80 for 280 elderly people aged 60 and over. Zanella et al. (29) culturally adapted the nutrition literacy scale to Brazilian Portuguese in 1,197 adults and reported that the new version of the 28-item nutrition literacy scale consists of 23 items and is a valid and reliable measurement tool.

While previous studies used the 28-item nutrition literacy scale developed by Diamond (10), the 11-item S-NutLit was developed by Vrinten et al. (1) depending on the dynamic structure of science. S-NutLit is a useful, valid, and reliable measurement tool that can determine nutrition literacy in a shorter time and with fewer questions, with 11 items instead of 28 items. Chilón-Troncos (30) adapted the S-NutLit scale for 396 Peruvian adults. According to this result, a one-dimensional analysis of 11 items was conducted to explain a single factor to verify the fit of the model, and they reported that the single-factor model did not achieve a good fit. On the other hand, they reported that the two-factor model, namely IS (*α* = 0.83) and ES (*α* = 0.79), was the most appropriate model for the S-NutLit scale. In our study, the Cronbach’s alpha coefficient for the S-NutLit-Tr scale was obtained as 0.86. In addition, it was found to be 0.84 and 0.77 for the IS and ES sub-dimensions, respectively.

As with many studies, this study had some limitations. The most important limitation of the research was the collection of data using the convenience sampling method. Nevertheless, the sample of this study was adequate and the results for the S-NutLit-Tr were valid and reliable. In addition to these limitations, the S-NutLit-Tr was found to be highly valid and reliable under the applied conditions, and the effectiveness of using the scale in further research was demonstrated. However, it is important that this study can be applied to a larger population in Turkey (students at various universities, different ethnicities, different socio-economic groups, etc.) so that the generalizability of the scale can be repeated. Thus, a cutoff score can be determined to evaluate the sensitivity of S-NutLit-Tr and distinguish between high and low nutrition literacy.

As a result of this study, researchers and health promotion professionals can use S-NutLit-Tr to monitor nutrition literacy in young adults. Due to its brevity, S-NutLit-Tr may play a mediating role in assessing nutrition behavior/knowledge. The relationships between participants’ characteristics such as ethnicity, other demographic characteristics, health knowledge and status, and physical activity levels with the S-NutLit-Tr scale can be investigated in detail in future studies. Additionally, in future studies, differences and similarities in nutrition literacy levels between different countries can be examined in detail.

## Data availability statement

The raw data supporting the conclusions of this article will be made available by the authors, without undue reservation.

## Ethics statement

For this study, ethical permission was taken from the Istanbul Gelisim University Ethics Committee (Meeting no: 2024-03, Meeting date: 29.02.2024). The studies were conducted following the local legislation and institutional requirements. The participants provided their written informed consent to participate in this study.

## Author contributions

MCK: Conceptualization, Project administration, Writing – original draft. EY: Data curation, Formal analysis, Methodology, Writing – review & editing. OI: Data curation, Methodology, Writing – original draft, Writing – review & editing. PA: Writing – original draft, Writing – review & editing. L-GT: Methodology, Writing – original draft, Writing – review & editing. CZ: Writing – original draft, Writing – review & editing.

## Appendix

Genç Yetişkinler için Beslenme Okuryazarlığı Ölçeği Kısa Form (GYBOÖ-KF) Türkçe versiyonu

**Table:**

No	Maddeler
1.	Sağlıklı beslenme hakkında sorularım olursa, bu konuda nerede bilgi bulabileceğimi biliyorum.
2.	Sürdürülebilir beslenme örnekleri arasında organik sebzeler, serbest gezen tavuk yumurtaları, adil ticaret kahvesi vb. sayılabilir.
3.	Gıda üçgeninin temel kurallarına aşinayım.
4.	Beslenmeyle ilgili bilgilerin para kazanmak amacıyla, örneğin bir ürün satmak isteyen kişiler tarafından yazılıp yazılmadığını değerlendirebilirim.
5.	İnternette beslenme hakkında bilgi aradığımda, güvenilir ve daha az güvenilir web siteleri arasında ayrım yapabilirim.
6.	Reklamlar genellikle beslenme ve sağlık arasında bağlantı kurar. Bu bağlantıların doğru olup olmadığına kolayca karar verebilirim.
7.	Yemek pişirirken beslenme ile ilgili bilgileri uygulayabilmek için gerekli becerilere sahibim.
8.	Medyada yer alan beslenme ile ilgili bilgilerin güvenilir olup olmadığını değerlendirebilirim.
9.	Beslenme ile ilgili bilgileri uzmanlarla tartışırım.
10.	Uzmanlardan gelen beslenme tavsiyelerine uyarım.
11.	Beslenme alışkanlığımı güncel bilimsel bilgilere dayandırırım.
